# Hypercalcemia, metabolic alkalosis and renal failure secondary to calcium bicarbonate intake for osteoporosis prevention - ‘modern’ milk alkali syndrome: a case report

**DOI:** 10.4076/1757-1626-2-6188

**Published:** 2009-08-06

**Authors:** Alain Waked, Abdallah Geara, Badiaa El-Imad

**Affiliations:** Department of Medicine, Staten Island University Hospital27 Hickory AvenueStaten IslandNY 10305, USA

## Abstract

We report a case of a patient presenting with a triad of hypercalcemia, metabolic alkalosis and renal failure secondary to calcium bicarbonate intake for osteoporosis prevention. It is the classical presentation of the “modern” milk alkali syndrome that presents several characteristics distinguishing it from the “old” syndrome described secondary to peptic ulcer disease treatment. Milk alkali syndrome affects middle-aged female patients taking over-the-counter calcium carbonate. Clinically, these patients present in an acute hypercalcemia crisis, responding rapidly to hydration. The phosphorus level is normal to low. Bisphosphonate should be used cautiously due to the risk of symptomatic hypocalcemia.

## Introduction

In 1912, Sippy described a “cure” for gastric ulcer consisting of milk and antacid (calcium carbonate, sodium bicarbonate, magnesium oxide and bismuth subcarbonate) [1]. During the following three decades, several case reports of complications secondary to the high calcium and bicarbonate content of this solution lead to the description of three clinical “milk alkali syndrome” (MAS) [2,3]: acute, subacute (Cope’s syndrome) and chronic (Brunett’s syndrome) [4]. After the advent of non absorbable antacids and later histamine-2 blockers, the incidence of this syndrome dropped to less than 1% of etiologies of hypercalcemia in the mid-seventies [5].

During the last 2 decades, cases of MAS have been reported frequently and the authors suggested that this syndrome is reemerging (incidence of MAS as a cause of hypercalcemia was reported in new series to be as high as 12%, the third leading cause of hypercalcemia) [6,7]. The epidemiology and the etiology of MAS changed. We report a case of this “modern version” of MAS.

## Case presentation

Our patient is an 81-year-old white male, citizen of United States, who presented to our hospital for a one week history of lethargy and nausea. The day of admission, he developed an acute change in mental status, and became disoriented, confused and somnolent. The patient was known to have hypertension, diabetes mellitus and benign prostate hypertrophy. On physical examination, the patient was severely cachectic and disoriented. His vital signs were in the normal range except for a high blood pressure of 200/100 mmhg.

The initial blood tests on admission showed a creatinine of 8.3 mg/dl and a serum calcium concentration of 14.6 mg/dl. Two months previously the patient had a creatinine of 1.2 mg/dl and a serum calcium concentration of 8.9 mg/dl. In addition, the patient presented with metabolic alkalosis. The admission diagnosis was hypercalcemia leading to acute renal failure and contraction metabolic alkalosis.

After aggressive saline hydration for 2 days, the patient regained his baseline level of consciousness, the serum calcium concentration returned to normal values. However, the glomerular filtration rate did not recover and was still less than 10 ml/min. The patient was started on hemodialysis for 2 sessions and then stopped upon the patient’s request to stop all additional therapy and he opted for hospice care. A repeat laboratory test in four weeks after his hospital discharged showed a glomerular filtration rate of 47 ml/min and a serum creatinine of 1.2 mg/dl implying a spontaneous resolution of his acute renal failure (Table 1).

**Table 1.** The case happened in Staten Island University Hospital.

	Admission	2 days	7 days	1 month after discharge
Calcium (mg/dl)	13.8	10.1	8.4	8.3
Phosphorus (mg/dl)	5.7	5.5	4	
Albumin (g/dl)	1.9		2.3	2.4
BUN (mg/dl)	112	98	72	25
Crea (mg/dl)	8.3	7.8	6.2	1.5
GFR	6	7	9.1	47
Na (meq/l)	137	129	136	135
K (meq/l)	5.6	4.9	3.7	4
Cl (mg/l)	97	96	101	103
Bicarbonate (mg/l)	37	22	24	23
Hemoglobin (g/dl)	11.8	9.1	8.2	
Hematocrit (%)	33	30.1	23.9	
pH	7.67			
PTH (pg/ml)	11.3			

The diagnosis was established on a repeat interview with the patient after he regained his orientation in which he admitted taking an estimate of twenty five tablets of calcium carbonate every day as a self medication for osteoporosis prevention. Our final diagnosis is hypercalcemia secondary to milk alkali syndrome.

## Discussion

MAS consists of a triad of hypercalcemia, metabolic alkalosis and renal insufficiency associated with the ingestion of large amounts of calcium and absorbable alkali. Classically described as secondary to treatment of peptic ulcer disease with Sippy’s regimen, in the modern version of milk alkali syndrome the source of calcium is usually calcium carbonate given for several indications (osteoporosis treatment and prevention, phosphate binder in renal failure, with glucocorticoid therapy). In the Far East, Asia and South Pacific, MAS could be induced by betel nut chewing [8,9].

MAS has changed from a middle-aged male dominated condition [10] to one with more than half of the patients being female of an average age of 50 years [11]. The diagnosis is based on a history of ingestion of calcium rich compounds, concordant biochemical findings, and exclusion of other causes especially primary hyperparathyroidism and hypercalcemia of malignancy. The reported intake of calcium bicarbonate ranges from 2.5 grams (5 tablets) to 20 grams per day. Parathyroid hormone (PTH) levels are suppressed and undergo a rapid rebound increase within hours of the decrease in serum calcium and peaks approximately 1 week later with a subsequent return to normal levels [6,11].

At the level of the biochemical markers, MAS patients used to present with increased in serum phosphorus values due to the ingestion of phosphorus-rich milk together with the suppression of PTH that leads to reduced phosphorus excretion. MAS secondary to calcium carbonate supplementation could present with normal to low serum phosphorus values. In these patients, there is no phosphorus supplementation and the calcium carbonate acts as phosphate binder [12,13]. The presentation in 50 percent of patients is acute and symptomatic hypercalcemia. In the chronic form, MAS is usually asymptomatic, with incidental finding of hypercalcemia and renal failure.

The complications associated with milk alkali syndrome are metastatic calcifications, pancreatitis and reversible cardiac conduction abnormalities [14-16]. As for the management, withdrawal of the offending agent and intravenous volume expansion are the most important initial steps. Usually, these interventions will reverse the hypercalcemia and the alkalosis. Renal function could return to normal if the diagnosis of MAS is made early in the course of the disease. Hemodialysis may be required in some clinical settings. In patients presenting with very high serum calcium levels, the addition of furosemide, pamidronate and hydrocortisone may be helpful. Bisphosphonates should be used cautiously in MAS since bone resorption is not thought to play an important role in the pathogenesis of this syndrome, and the usage of bisphosphonates has been complicated in several cases by symptomatic hypocalcemia requiring intravenous calcium supplementation even in cases with initially very high serum calcium levels [17].

## Conclusion

Although MAS has been frequently reported in the recent medical literatures (more than 54 patients have been reported from 1983 through 2004), this syndrome remains under diagnosed due to several reasons. First, it is occurring in a wider medical spectrum than the former association with the treatment of peptic ulcer disease. Second, the incidence is underestimated in medical practice. Third, the question of over-the-counter medications is not frequently asked during a medical encounter.

Although, our case report does not seem interesting as far as rarity, we opted to write in order to increase the awareness over the reemergence of MAS, especially that its under diagnosis was obvious in the case reports published (three of these 54 patients underwent parathyroid gland exploration, 20 had permanent renal function impairment) [11]. Given that calcium has replaced milk products as main source of calcium, we find that it could be beneficial to give a new nomenclature for this “modern version” of MAS. “Calcium alkali syndrome”, as suggested by kaklamanos et al. [18], would be an appropriate name. This new nomenclature could lead to an increase in awareness of this diagnosis and underlines the differences between the “Old” and the “New” MAS.

## Abbreviations

MAS: Milk alkali syndrome
PTH: Parathyroid hormone
